# Clinical evaluation of seven tumour markers in lung cancer diagnosis: can any combination improve the results?

**DOI:** 10.1038/bjc.1995.296

**Published:** 1995-07

**Authors:** M. Plebani, D. Basso, F. Navaglia, M. De Paoli, A. Tommasini, A. Cipriani

**Affiliations:** Department of Laboratory Medicine, University of Padova, Italy.

## Abstract

In this study we compared the diagnostic utility of: (1) neuron-specific enolase (NSE); (2) squamous cell carcinoma antigen (SCC); (3) carcinoembryonic antigen (CEA); and (4) cytokeratin markers (CYFRA 21-1, TPA, TPM, TPS) in patients with small-cell lung cancer (SCLC) (21 cases) and non-small-cell lung cancer (94 cases). For comparison we also studied 66 patients with benign lung diseases and nine with pleural mesothelioma. NSE levels in SCLC patients were significantly higher than those in all the other groups studied. No significant variations were found among the SCC levels in all groups. CEA levels in patients with adenocarcinoma were significantly higher than those in all other groups studied. CYFRA 21-1 serum levels significantly increased in patients with squamous cell carcinoma and mesothelioma, while TPA, TPS and TPM increased in patients with lung cancer irrespective of the histological type. In patients with SCLC, high levels of all markers except SCC were found when the disease was extensive. In patients with non-SCLC, the highest levels of all tumour markers were usually found in those with advanced disease, although CYFRA 21-1 gave a sensitivity of 44% when a specificity of 95% was fixed in stage I non-SCLC patients. An analysis of receiver operating characteristic curves revealed that the highest diagnostic accuracies in distinguishing benign from malignant lung diseases were achieved with TPM (81%), CYFRA 21-1 (72%), CEA (78%) or TPA (78%) when using cut-off values of 46 Ul-1, 3.0 micrograms l-1, 2.0 micrograms l-1 and 75 Ul-1 respectively. When all patients were considered, the combined evaluation of more than one marker did not significantly improve the results obtained with TPM alone. However, taking into consideration the fact that CYFRA 21-1 is the most sensitive index of early lung tumours and that its combined determination with TPM did not worsen the overall sensitivity and specificity of the latter, the combined use of these two markers may be suggested as a useful took for the diagnosis of lung tumours.


British Journal of Cancer (1995) 72, 170-173

© 1995 Stockton Press All rights reserved 0007-0920/95 $12.00

### Clinical evaluation of seven tumour markers in lung cancer diagnosis: can any combination improve the results?

M Plebani[1,2], D Basso[1], F Navaglia[1], M De Paoli[1], A Tommasini[3] and A Cipriani[3]

[1]*Department of Laboratory Medicine, University of Padova;* [2]*Centre of Biomedical Research, Castelfranco Veneto;* [3]*Department of Pneumology, University of Padova, Italy.*

**Summary** In this study we compared the diagnostic utility of: (1) neuron-specific enolase (NSE); (2) squamous cell carcinoma antigen (SCC); (3) carcinoembryonic antigen (CEA); and (4) cytokeratin markers (CYFRA 21-1, TPA, TPM, TPS) in patients with small-cell lung cancer (SCLC) (21 cases) and non-small-cell lung cancer (94 cases). For comparison we also studied 66 patients with benign lung diseases and nine with pleural mesothelioma. NSE levels in SCLC patients were significantly higher than those in all the other groups studied. No significant variations were found among the SCC levels in all groups. CEA levels in patients with adenocarcinoma were significantly higher than those in all other groups studied. CYFRA 21-1 serum levels significantly increased in patients with squamous cell carcinoma and mesothelioma, while TPA, TPS and TPM increased in patients with lung cancer irrespective of the histological type. In patients with SCLC, high levels of all markers except SCC were found when the disease was extensive. In patients with non-SCLC, the highest levels of all tumour markers were usually found in those with advanced disease, although CYFRA 21-1 gave a sensitivity of 44% when a specificity of 95% was fixed in stage I non-SCLC patients. An analysis of receiver operating characteristic curves revealed that the highest diagnostic accuracies in distinguishing benign from malignant lung diseases were achieved with TPM (81%), CYFRA 21-1 (72%), CEA (78%) or TPA (78%) when using cut-off values of $46\ U\ l^{-1}$, $3.0\ \mu g\ l^{-1}$, $2.0\ \mu g\ l^{-1}$ and $75\ U\ l^{-1}$ respectively. When all patients were considered, the combined evaluation of more than one marker did not significantly improve the results obtained with TPM alone. However, taking into consideration the fact that CYFRA 21-1 is the most sensitive index of early lung tumours and that its combined determination with TPM did not worsen the overall sensitivity and specificity of the latter, the combined use of these two markers may be suggested as a useful took for the diagnosis of lung tumours.

**Keywords:** lung cancer; CEA; CYFRA 21-1; NSE; TPA; SCC

Lung cancer, the most frequent malignant tumour in industrialised countries after breast and colorectal cancer, is classified as small-cell lung cancer (SCLC), squamous cell carcinoma, primary adenocarcinoma and large-cell carcinoma. The neuroendocrine properties of SCLC give it specific biological and clinical features. Since from a prognostic and therapeutic viewpoint squamous cell carcinoma, primary adenocarcinoma and large-cell carcinoma behave similarly, they are all pooled into a single group named non-small-cell lung cancer (Carney and De Leij, 1988).

In order to improve the clinical approach to lung cancer patients, several serum tumour markers have been studied, and in this context recent interest has been focused on the role of serum cytokeratins (Pujol *et al.*, 1993; Stieber *et al.*, 1993*a,b*; Ebert *et al.*, 1994; Plebani *et al.*, 1994; van der Gaast *et al.*, 1994), which make up the intermediate filament cytoskeleton within epithelial cells, and consist of at least 19 different polypeptides, numbered 1 to 19 (Lazarides, 1980; Moll *et al.*, 1982).

Cytokeratins can be detected both in normal as well as in malignant tissues of epithelial origin (Moll *et al.*, 1982; Broers *et al.*, 1988), and each polypeptide seems to be typical of certain types of epithelial differentiation (Moll *et al.*, 1982; Sun *et al.*, 1983).

Tissue polypeptide antigen (TPA) detects mainly cytokeratins 8 and 19 and, to a very small degree, cytokeratin 18, while tissue polypeptide-specific antigen (TPS) mainly detects cytokeratin 18 and, to a small extent, cytokeratins 8 and 19 (Bodenmuller, 1993). TPA is recognised by polyclonal antisera against cytokeratins 8, 18 and 19. To enhance the sensitivity and specificity of TPA assay, a new assay is now available, which is based on the use of three monoclonal antibodies directed against cytokeratins 8, 18 and 19, allowing detection of TPM antigen (Gion *et al.*, 1994). CYFRA 21-1 detects a fragment of cytokeratin 19 (Pujol *et al.*, 1993). Of these markers, TPA and CYFRA 21-1 seem to be the most sensitive and specific serum markers of lung cancer (Mizushima *et al.*, 1991; Stieber *et al.*, 1993*a*; Ebert *et al.*, 1994).

The neuroendocrine marker serum neuron-specific enolase (NSE) is helpful in the diagnosis and monitoring of SCLC (Harding *et al.*, 1990; Jorgensen *et al.*, 1992; Bergman *et al.*, 1993). Squamous cell carcinoma antigen (SCC) and carcinoembryonic antigen (CEA) have been extensively studied in patients with lung cancer; however, their sensitivity and specificity have usually been reported as low (Mizushima *et al.*, 1991; Jarvisalo *et al.*, 1993; Ebert *et al.*, 1994). Few reports deal with a combined evaluation of various tumour markers in lung cancer diagnosis (Mizushima *et al.*, 1991; Jarvisalo *et al.*, 1993; Stieber *et al.*, 1993*a*; Ebert *et al.*, 1994).

The aims of this study were therefore to: (1) assess the behaviour of CYFRA 21-1, TPA, TPS, TPM, NSE, SCC and CEA in patients with SCLC and with non-small-cell lung cancer; (2) evaluate whether their combined determination enhances their diagnostic accuracy; and (3) assess whether there is any correlation between tumour stage and serum marker levels.

### Materials and methods

We studied a total of 190 subjects: 66 (42 males, 24 females, age range 19-70) had benign lung disease (BLD, Table I); 115 (103 males, 12 females, age range 45-74) had histologically confirmed lung cancer (54 squamous cell carcinoma, 40 adenocarcinoma and 21 small-cell lung cancer); nine males had pleural mesothelioma (age range 50-65). The patients with SCLC were subdivided on the basis of limited ($n = 10$) or extensive ($n = 11$) tumour spread. Non-small-cell lung cancer was classified as: stage I ($n = 18$), stage II

Correspondence: M Plebani, Istituto di Medicina di Laboratorio, c/o Laboratorio Centrale, Via Giustiniani 2, 35128 Padova, Italy
Received 26 July 1994; revised 16 January 1995; accepted 8 February 1995

(*n* = 15), stage III (*n* = 25) and stage IV (*n* = 36). After overnight fasting, at diagnosis, a serum sample was obtained from each subject. The sera were kept at −20°C for no more than 1 month until the biochemical determinations were made. The following parameters were measured: CYFRA 21-1 (ELISA, Enzymun-test, Boehringer Mannheim, Italy); TPA, TPM, NSE (Byk Gulden, Italy), TPS (Medical System, Italy), SCC (Abbott, USA) and CEA (Sorin Biomedica, Italy).

A statistical analysis of the results was made using the analysis of variance (ANOVA, one-way), Bonferroni's test for pairwise comparisons (Guenther, 1964), Student's *t*-test and receiver operating characteristic (ROC) curves (Weinstein and Fineberg, 1980).

**Table I** Benign lung diseases (BLD)

| | |
|---|---|
| Chronic obstructive pulmonary disease | 19 |
| Sarcoidosis | 14 |
| Bronchopneumonia | 10 |
| Chronic bronchitis | 7 |
| Pulmonary fibrosis | 2 |
| Tuberculosis | 4 |
| Asthma | 3 |
| Hamartoma | 2 |
| Pleuritis | 2 |
| Cystic fibrosis | 1 |
| Loffler's syndrome | 1 |
| Neurofibroma | 1 |
| Total | 66 |

**Figure 1** Individual values of serum CEA and CYFRA 21-1 in the material studied. BLD, benign lung disease; SQC, squamous cell carcinoma; ADE, lung adenocarcinoma; SCLC, small-cell lung cancer; MESO, pleural mesothelioma. CEA levels were significantly higher in patients with ADE as compared with BLD, SQC and SCLC (one-way ANOVA, $F = 4.84$, $P < 0.001$). CYFRA 21-1 levels were significantly higher in patients with SQC as compared with BLD and in patients with MESO as compared with BLD, SQC and SCLC (one-way ANOVA, $F = 4.27$, $P < 0.005$). Triangle = ten patients.

### Results

Figure 1 shows the individual values for CYFRA 21-1 and of CEA in the studied material; the results of the analysis of variance and Bonferroni's test are reported in the figure legend.

Table II reports mean values, standard errors of the means and findings from the statistical analysis of TPA, TPS, TPM, NSE and of SCC in the different patient groups. Irrespective of the histological type, patients with lung cancer had significantly higher TPA, TPS and TPM than patients with benign lung diseases. In contrast, the NSE levels in patients with SCLC were significantly higher than those in all the other groups studied. No significant variations were found among the SCC levels of all groups.

Table III illustrates the sensitivity of the seven serum markers studied when a specificity of 95% was fixed; the corresponding cut-off values are also reported.

Figure 2 reports the sensitivity values, for a fixed specificity of 95%, of all markers in relation to tumour stage and histological type. For non-small-cell lung cancer the TNM classification was considered, while SCLC patients were subdivided on the basis of a limited or extensive disease.

Figure 3 illustrates the ROC curves of each marker in differentiating benign from malignant lung diseases. An analysis of the ROC curves revealed that the higher diagnostic accuracies were achieved with TPM (81%, sensitivity 79% and specificity 84%), CYFRA 21-1 (72%, sensitivity 64% and specificity 86%), CEA (78%, sensitivity 89% and specificity 56%) and TPA (78%, sensitivity 82% and specificity 71%) when using cut-off values of 46 U $l^{-1}$, 3.0 μg $l^{-1}$, 2.0 μg $l^{-1}$ and 75 U $l^{-1}$ respectively.

Table IV reports sensitivity and specificity with the combined evaluation of CYFRA 21-1, CEA, TPA and TPM. The highest sensitivity was obtained when three markers were combined. This increase in sensitivity was achieved at the cost of specificity. The best specificity, with an overall satisfactory sensitivity, was obtained with the combination of CYFRA 21-1 and TPM.

### Discussion

All the cytokeratins we studied exhibited a similar pattern, being significantly higher in patients with any histological lung cancer type than in patients with benign lung diseases. As with the other cytokeratins, CYFRA 21-1 was not related to the histological tumour type. In contrast, CEA and NSE levels were higher in patients with adenocarcinoma and with SCLC respectively, than in patients with other lung tumours. Patients with squamous cell carcinoma had the highest SCC values, although this difference was not of statistical significance. When, however, a specificity of 95% was fixed, CYFRA 21-1, TPM and TPA in that order were found to be the most sensitive index in diagnosing lung cancer, confirming for CYFRA 21-1 the observations made by Stieber *et al.* (1993*a*) and Ebert *et al.* (1994). All the other markers had sensitivities below 35% and therefore could not be considered singly as useful markers of different types of lung cancer.

One of the main drawbacks of serum tumour markers is that the highest levels are usually found when disease is at an advanced stage. We therefore investigated whether there is any correlation between the markers studied and tumour stage. Patients with SCLC were subdivided into two groups on the basis of (1) tumour confined to one hemithorax, with or without mediastinal lymph node involvement (limited) or (2) tumour spread beyond this limit (extensive). Non-small-cell lung cancers were staged according to the TNM classification. In SCLC, all markers but SCC reached high levels in about 50% of patients with an extensive disease, while patients with limited SCLC were discriminated from patients with benign lung diseases in a very small percentage of cases. Therefore, none of the tumour markers studied can be considered an early indicator of this type of lung cancer.

Table II Mean values, standard errors of the means and statistical analysis of TPA, TPS, TPM, NSE and SCC in the different patient groups

| | TPA mean | ($U\,l^{-1}$) s.e.m. | TPS Mean | ($U\,l^{-1}$) s.e.m. | TPM Mean | ($U\,l^{-1}$) s.e.m. | NSE Mean | ($\mu g\,ml^{-1}$) s.e.m. | SCC Mean | ($\mu g\,l^{-1}$) s.e.m. |
|---|---|---|---|---|---|---|---|---|---|---|
| BLD | 68 | 8 | 102 | 12 | 29 | 4 | 12 | 1 | 1.2 | 0.13 |
| SQC | 211* | 28 | 193 | 44 | 152* | 23 | 14 | 1 | 3.3 | 0.64 |
| ADE | 223* | 43 | 320* | 89 | 162* | 32 | 14 | 2 | 5.3 | 3.72 |
| SCLC | 252* | 83 | 335* | 124 | 200* | 73 | 67† | 24 | 1.1 | 0.34 |
| MESO | 876† | 491 | 458* | 166 | 422† | 129 | 17 | 2 | 1.0 | 0.24 |
| ANOVA, one way | | | | | | | | | | |
| *F* | 9.695 | | 3.583 | | 10.709 | | 9.879 | | 1.033 | |
| *P*< | 0.001 | | 0.01 | | 0.001 | | 0.001 | | NS | |

*$P<0.05$ as compared to BLD and †$P<0.001$ as compared with all the other groups. (Bonferroni's test for pairwise comparisons). BLD, benign lung disease; SQC, squamous cell carcinoma; ADE, lung adenocarcinoma; SCLC, small-cell lung cancer; MESO, pleural mesothelomia.

Table III Sensitivity of CYFRA 21-1, CEA, NSE, SCC, TPA, TPM and TPS in diagnosing lung cancer when a specificity of 95% was fixed. The corresponding cut-off values are also reported

| *Tumour marker* | *Sensitivity (%)* | *Cut-off* |
|---|---|---|
| CYFRA 21-1 | 52 | $4\,\mu g\,l^{-1}$ |
| TPM | 47 | $100\,U\,l^{-1}$ |
| TPA | 43 | $150\,U\,l^{-1}$ |
| CEA | 35 | $7\,\mu g\,l^{-1}$ |
| TPS | 25 | $300\,U\,l^{-1}$ |
| NSE | 23 | $22\,\mu g\,l^{-1}$ |
| SCC | 15 | $4\,\mu g\,l^{-1}$ |

Figure 2 Sensitivities of CYFRA 21-1, TPM, TPA, CEA, TPS, NSE and SCC in diagnosing lung cancer on the basis of tumour stage and histology. To calculate sensitivity, a fixed specificity of 95% was considered. For non-small-cell lung cancer (a) the TNM classification was considered, while patients with small-cell lung cancer (b) were subdivided on the basis of a limited or extensive disease.

Table IV Combined evaluation of CYFRA 21-1, CEA, TPA and TPM: sensitivity in diagnosing lung cancer ($n = 124$) and specificity *vs* benign lung diseases ($n = 66$)

| *Markers* | *Sensitivity (%)* | *Specificity (%)* |
|---|---|---|
| CYFRA 21-1 or CEA | 87 | 49 |
| CYFRA 21-1 or TPA | 85 | 70 |
| CYFRA 21-1 or TPM | 79 | 79 |
| CYFRA 21-1 or CEA or TPA | 94 | 41 |
| CYFRA 21-1 or CEA or TPM | 92 | 42 |

Cut-off values were $3.0\,\mu g\,l^{-1}$, $2.0\,\mu g\,l^{-1}$, $75\,U\,l^{-1}$ and $46\,U\,l^{-1}$ for CYFRA 21-1, CEA, TPA and TPM respectively. Positive patients were considered to be those having at least one marker above the cut-off, while negative patients were considered to be those having all markers below the cut-off.

Figure 3 Receiver operating characteristic (ROC) curves of the seven serum markers studied in distinguishing patients with lung cancer from patients with benign lung diseases.

The most sensitive indicator of patients with non-small-cell lung cancer (stage I) was CYFRA 21-1 (44%). More advanced stages of these tumours caused increased levels of CYFRA 21-1 as well as of TPM, TPA and CEA, but not of NSE and SCC.

In order to improve upon the results obtained with each serum marker, we ascertained their sensitivity and specificity in the diagnosis of lung cancer when evaluated together. This was done using TPM, CYFRA 21-1, TPA and CEA. These markers were chosen because ROC curve analysis indicated that they had the highest diagnostic accuracies (81%, 72%, 78% and 78% for cut-off values of $46\,U\,l^{-1}$, $3.0\,\mu g\,l^{-1}$, $75\,U\,l^{-1}$ and $2.0\,\mu g\,l^{-1}$ respectively). When the combination of three markers was considered, a significant increase in sensitivity was observed, in spite of the fact that the specificity never exceeded 45%. Similar results were obtained when CYFRA 21-1 was combined with CEA or TPA. The combined evaluation of TPM and CYFRA 21-1 merits special comment. The combination of these two markers did not significantly modify the sensitivity and specificity of TPM alone. However, CYFRA 21-1 determination should be recommended since it gave the most reliable results in identifying early non-small-cell lung tumours.

A question that might arise is which cut-off value should be considered: the one giving a specificity of 95% or the one giving the best combination of sensitivity and specificity and therefore the highest accuracy? We suggest the latter in the first instance when lung cancer is suspected and the former when a more clear-cut distinction between benign and malignant lung diseases is required. Thus, two different decision levels can be suggested in relation to the clinical use of tumour markers: as a first step in the diagnostic algorithm or when a differentiation from benign lung diseases is required.

In conclusion, the use of tumour markers can be of some help in the diagnosis of small-cell and non-small-cell lung cancer; cytokeratins in serum are increased independently of histological tumour type. Overall, the most sensitive and specific indices of lung cancer are TPM and CYFRA 21-1; their combined use is to be recommended, since the latter was found in 44% of cases of early tumour, while the former was found mainly in patients with advanced lung tumours.

**Acknowledgements**

This study was conducted under the auspices of the Veneto Region, Grant No. 114-05-1985 'Valutazione dell'efficienza diagnostica di marcatori biochimici tumorali'.

**References**

BERGMAN B, BREZICKA F-T, ENGSTROM C-P AND LARSSON S. (1993). Clinical usefulness of serum assays of neuron-specific enolase, carcinoembryonic antigen and CA-50 antigen in the diagnosis of lung cancer. *Eur. J. Cancer*, **29A**, 198-202.

BODENMULLER H. (1993). Symposium 'Cytokeratins and tissue polypeptide antigen' 18-19 March 1993, Istituto Nazionale per lo Studio e la Cura dei Tumori, Milano. *Int. J. Biol. Markers* (in press).

BROERS JL, RAMAEKERS FC, ROT MK, OOSTENDORPT, HUYSMANS A, VAN MUIJEN GN, WAGENAAR SS AND VOOIJS GP. (1988). Cytokeratins in different types of human lung cancer as monitored by chain-specific monoclonal antibodies. *Cancer Res.*, **48**, 3221-3229.

CARNEY DN AND LEIJ LD. (1988). Lung cancer biology. *Semin. Oncol.*, **15**, 199-214.

EBERT W, DIENEMANN H, FATEH-MOGHADAM A, SCHEULEN M, KONIETZKO N, SCHLEICH T AND BOMBARDIEREI E. (1994). Cytokeratin 19 fragment CYFRA 21-1 compared with carcinoembryonic antigen, squamous cell carcinoma antigen and neuron-specific enolase in lung cancer. *Eur. J. Clin. Chem. Clin. Biochem.*, **32**, 189-199.

GION, M., MIONE R, BARIOLI P, SARTORELLO P AND CAPITANIO G. (1994). Tissue polypeptide antigen and tissue polypeptide specific antigen in primary breast cancer. Evaluation in serum and tumour tissue. *Eur. J. Clin. Chem. Clin. Biochem.*, **32**, 779-787.

GUENTHER WC. (1964). *Analysis of Variance*. Prentice-Hall: Englewood Cliff, NJ.

HARDING M, MCALLISTER J, HULKS G, VERNON D, MONIE R, PAUL J AND KAYE SB. (1990). Neurone specific enolase (NSE) in small cell lung cancer: a tumour marker of prognostic significance? *Br. J. Cancer*, **61**, 605-607.

JARVISALO J, HAKAMA M, KNEKT P, STENMAN UH, LEINO A, TEPPO L, MAATELA J AND AROMAA A. (1993). Serum tumor markers CEA, CA 50, TATI, and NSE in lung cancer screening. *Cancer*, **71**, 1982-1988.

JORGENSEN LGM, OSTERLIND K, HANSEN HH AND COOPER EH. (1992). Serum neuron specific enolase (NSE) is a determinant of response duration in small cell lung cancer (SCLC). *Br. J. Cancer*, **66**, 594-598.

LAZARIDES E. (1980). Intermediate filaments as mechanical integrators of cellular space. *Nature*, **283**, 249-256.

MIZUSHIMA Y, TSUJI H, IZUMI S, HIRATA H, KIN Y, KAWASAKI A, MATSUI S AND YANO S. (1991). Clinical evaluation of five tumor marker assays in patients with lung cancer. *Anticancer Res.*, **11**, 91-96.

MOLL R, FRANKE W, SHILLER DL, GEIGER B AND KREPLER R. (1982). The catalog of human cytokeratins: patterns of expression in normal epithelia, tumors and cultured cells. *Cell*, **31**, 11-24.

PLEBANI M, NAVAGLIA F, BASSO D, GIACOMINI A, CIPRIANI A, FACCHINETTI F, BORTOLOTTI A, DEL FAVERO G AND BURLINA A. (1994). Serum CYFRA 21-1 in the assessment of epithelial cancers. *J. Tumor Marker Oncol.*, **9**, 13-17.

PUJOL J-L, GRENIER J, DAURES J-P, DAVER A, PUJOL H AND MICHEL F-B. (1993). Serum fragment of cytokeratin subunit 19 measured by CYFRA 21-1 immunoradiometric assay as a marker of lung cancer. *Cancer Res.*, **53**, 61-66.

STIEBER P, DIENEMANN H, HASHOLZNER U, MULLER C, POLEY S, HOFMANN K AND FATEH-MOGHADAM A. (1993*a*). Comparison of cytokeratin fragment 19 (CYFRA 21-1), tissue polypeptide antigen (TPA) and tissue polypeptide specific antigen (TPS) as tumour markers in lung cancer. *Eur. J. Clin. Chem. Clin. Biochem.*, **31**, 689-694.

STIEBER P, HASHOLZNER U, BODENMULLER H, NAGEL D, SUNDER-PLASSMAN L, DIENEMANN H, MEIER W AND FATEH-MOGHADAM A. (1993*b*). CYFRA 21-1. A new marker in lung cancer. *Cancer*, **72**, 707-713.

SUN T-T, EICHNER R, NELSON WG, TSENG SCG, WEISS RA, JARVINEN M AND WOODCOCK-MITCHELL J. (1983). Keratin classes: molecular markers for different types of epithelial differentiation. *J Invest. Dermatol.*, **81**, 1095-1151.

VAN DER GAAST A, SCHOENMAKERS CHH, KOK TC, BLIJENBERG BG, CORNILLIE F AND SPLINTER TAW. (1994). Evaluation of a new tumour marker in patients with non-small-cell lung cancer: Cyfra 21.1. *Br. J. Cancer*, **69**, 525-528.

WEINSTEIN MC AND FINEBERG HV. (1980). *Clinical Decision Analysis*. WB Saunders: Philadelphia.


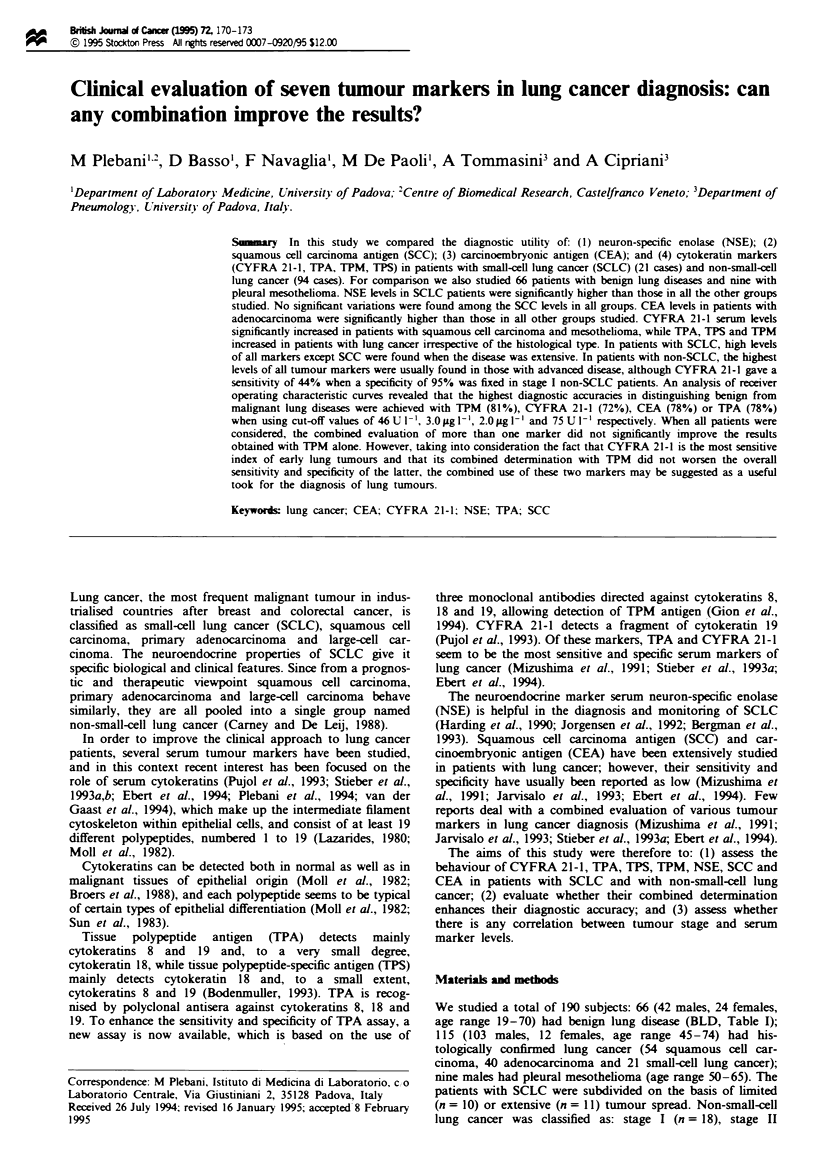

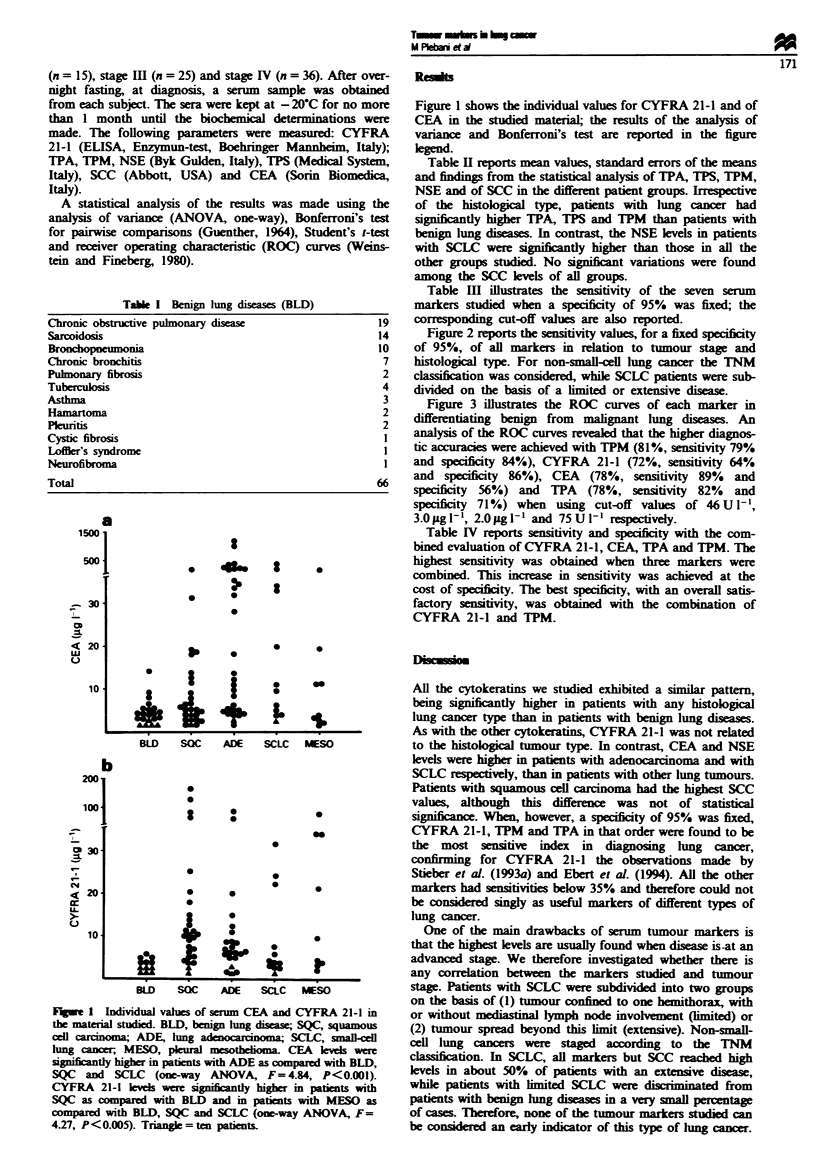

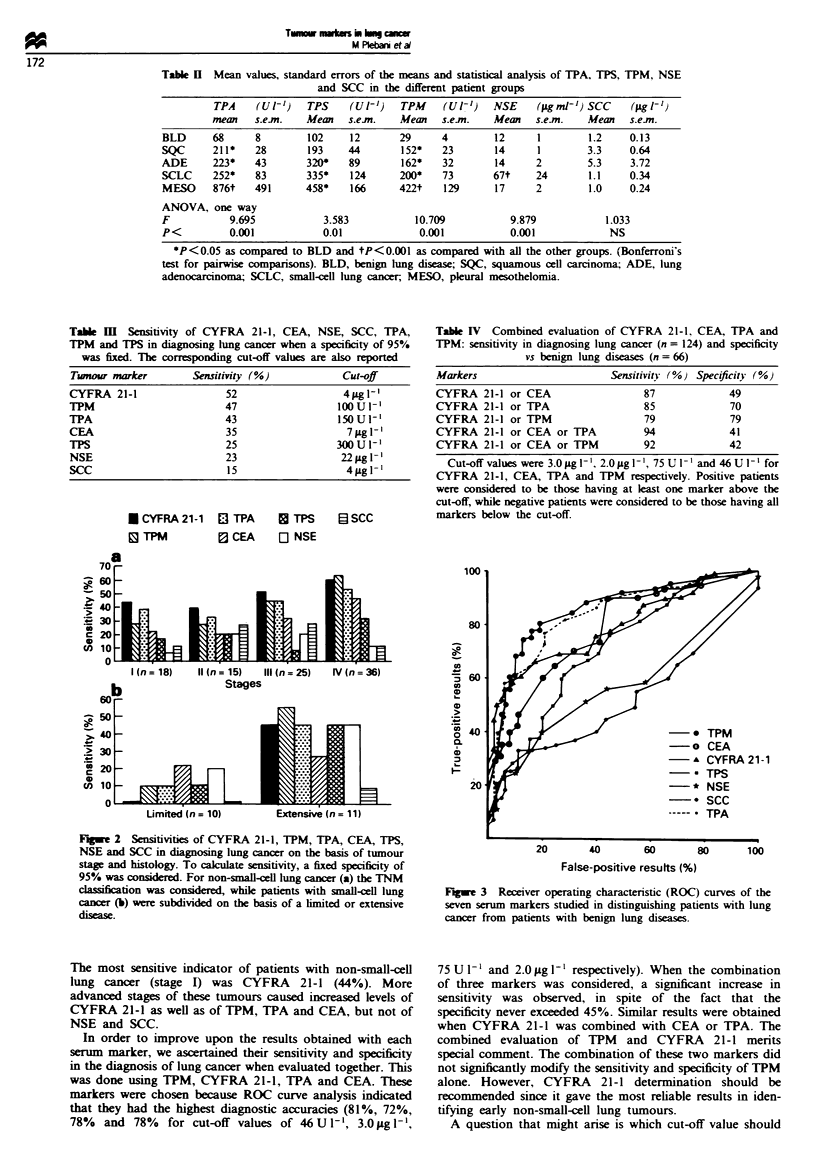

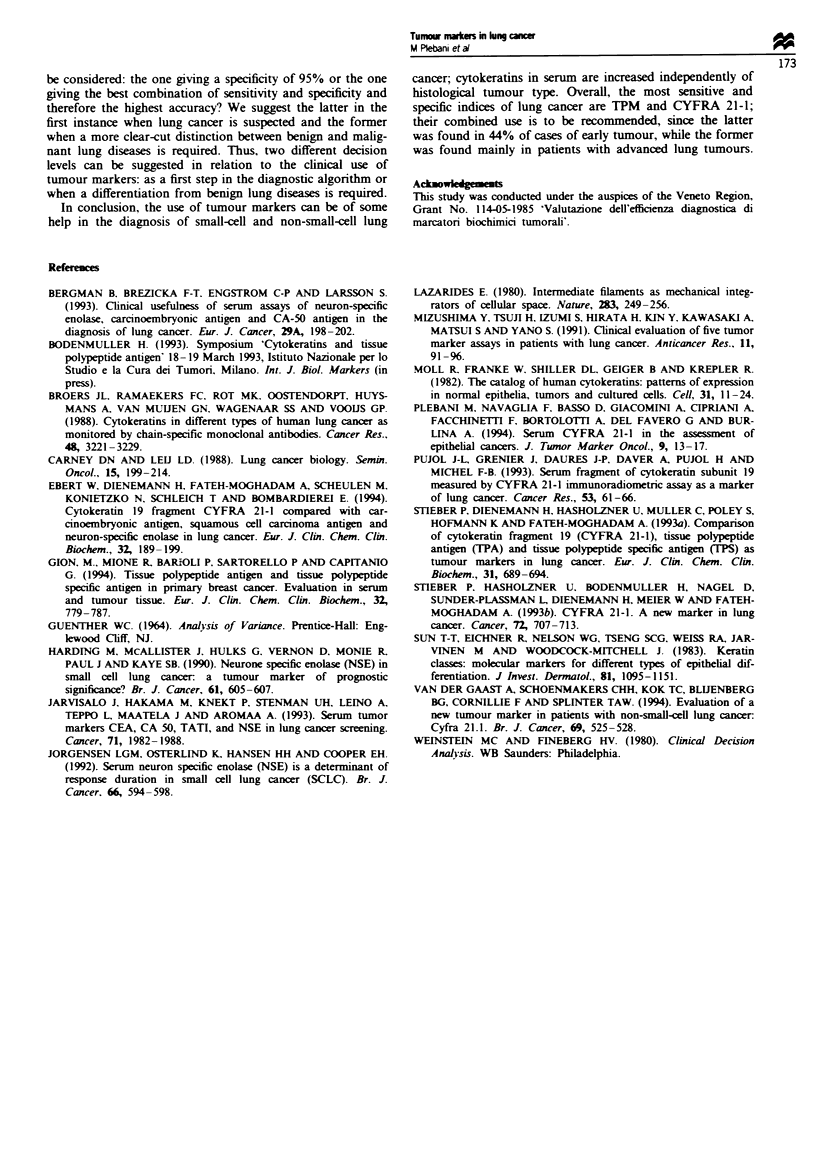


## References

[OCR_00482] Bergman B., Brezicka F. T., Engström C. P., Larsson S. (1993). Clinical usefulness of serum assays of neuron-specific enolase, carcinoembryonic antigen and CA-50 antigen in the diagnosis of lung cancer.. Eur J Cancer.

[OCR_00495] Broers J. L., Ramaekers F. C., Rot M. K., Oostendorp T., Huysmans A., van Muijen G. N., Wagenaar S. S., Vooijs G. P. (1988). Cytokeratins in different types of human lung cancer as monitored by chain-specific monoclonal antibodies.. Cancer Res.

[OCR_00501] Carney D. N., De Leij L. (1988). Lung cancer biology.. Semin Oncol.

[OCR_00506] Ebert W., Dienemann H., Fateh-Moghadam A., Scheulen M., Konietzko N., Schleich T., Bombardieri E. (1994). Cytokeratin 19 fragment CYFRA 21-1 compared with carcinoembryonic antigen, squamous cell carcinoma antigen and neuron-specific enolase in lung cancer. Results of an international multicentre study.. Eur J Clin Chem Clin Biochem.

[OCR_00513] Gion M., Mione R., Barioli P., Sartorello P., Capitanio G. (1994). Tissue polypeptide antigen and tissue polypeptide specific antigen in primary breast cancer. Evaluation in serum and tumour tissue.. Eur J Clin Chem Clin Biochem.

[OCR_00524] Harding M., McAllister J., Hulks G., Vernon D., Monie R., Paul J., Kaye S. B. (1990). Neurone specific enolase (NSE) in small cell lung cancer: a tumour marker of prognostic significance?. Br J Cancer.

[OCR_00528] Järvisalo J., Hakama M., Knekt P., Stenman U. H., Leino A., Teppo L., Maatela J., Aromaa A. (1993). Serum tumor markers CEA, CA 50, TATI, and NSE in lung cancer screening.. Cancer.

[OCR_00534] Jørgensen L. G., Osterlind K., Hansen H. H., Cooper E. H. (1992). Serum neuron specific enolase (NSE) is a determinant of response duration in small cell lung cancer (SCLC).. Br J Cancer.

[OCR_00540] Lazarides E. (1980). Intermediate filaments as mechanical integrators of cellular space.. Nature.

[OCR_00550] Moll R., Franke W. W., Schiller D. L., Geiger B., Krepler R. (1982). The catalog of human cytokeratins: patterns of expression in normal epithelia, tumors and cultured cells.. Cell.

[OCR_00560] Pujol J. L., Grenier J., Daurès J. P., Daver A., Pujol H., Michel F. B. (1993). Serum fragment of cytokeratin subunit 19 measured by CYFRA 21-1 immunoradiometric assay as a marker of lung cancer.. Cancer Res.

[OCR_00566] Stieber P., Dienemann H., Hasholzner U., Müller C., Poley S., Hofmann K., Fateh-Moghadam A. (1993). Comparison of cytokeratin fragment 19 (CYFRA 21-1), tissue polypeptide antigen (TPA) and tissue polypeptide specific antigen (TPS) as tumour markers in lung cancer.. Eur J Clin Chem Clin Biochem.

[OCR_00576] Stieber P., Hasholzner U., Bodenmüller H., Nagel D., Sunder-Plassmann L., Dienemann H., Meier W., Fateh-Moghadam A. (1993). CYFRA 21-1. A new marker in lung cancer.. Cancer.

[OCR_00586] van der Gaast A., Schoenmakers C. H., Kok T. C., Blijenberg B. G., Cornillie F., Splinter T. A. (1994). Evaluation of a new tumour marker in patients with non-small-cell lung cancer: Cyfra 21.1.. Br J Cancer.

